# Circular RNA circCOL6A3_030 is involved in the metastasis of gastric cancer by encoding polypeptide: circCOL6A3_030 promotes metastasis.

**DOI:** 10.1080/21655979.2021.1979915

**Published:** 2021-10-27

**Authors:** Xiaoge Geng, Jingya Wang, Chenjing Zhang, Xiaolu Zhou, Jiyong Jing, Wensheng Pan

**Affiliations:** a Department of Gastroenterology, Zhejiang Provincial People’s Hospital, People’s Hospital of Hangzhou Medical College, Hangzhou, Zhejiang, China; b Zhejiang Provincial People’s Hospital, People’s Hospital of Hangzhou Medical College, Hangzhou, Zhejiang, China

**Keywords:** Circcol6a3_030, metastasis, encoding, polypeptide, gastric cancer

## Abstract

Gastric cancer (GC) is a serious digestive tract disease that threatens human life worldwide, and the prognosis of gastric cancer accompanied by distant lymph node or the distant metastasis organs is worse. The purpose of this study was to investigate the role of circular RNA COL6A3_030 (circBase ID: hsa_circ_0006401; circRNADb ID: hsa_circ_28198; circBank ID: hsa_circCOL6A3_030) in GC metastasis. qRT-PCR analysis using back-splicing primers and Sanger sequencing of PCR products were performed to identify circCOL6A3_030 in GC tissues and cell lines; RNA-FISH assay was performed to validate the subcellular localization of circCOL6A3_030. Transwell and wound-healing assays were carried out to evaluate the migration ability of GC cells. Western blot was conducted to detect the polypeptide encoded by circCOL6A3_030 in cells. circCOL6A3_030 was down-regulated in GC tissues and cell lines, while circCOL6A3_030 was up-regulated in GC with distant lymph node metastasis. The migration of circCOL6A3_030 silenced GC cells was significantly inhibited in both SGC-7901 and BGC-823 cell lines. Importantly, in vivo assay, silencing circCOL6A3_030 could reduce liver metastases from gastric cancer cells. Meanwhile, further studies suggested that circCOL6A3_030 encoded a small peptide that had a function as a tumor-promoting metastasis factor and immunohistochemistry confirmed the presence of this polypeptide. To sum up, our study showed that circCOL6A3_030 promoted GC cell migration by encoding a small peptide called circCOL6A3_030_198aa. Therefore, our results highlight the potential role of circCOL6A3_030 for clinical diagnosis and treatment of GC with distant lymph node metastasis.

## Graphical abstract

**Figure:**
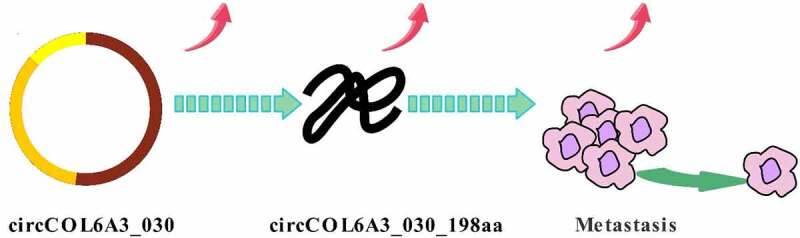

## Introduction

Gastric cancer (GC), also known as stomach adenocarcinoma, ranks as the fourth most common malignant tumor of the gastrointestinal in East Asia and the third leading cause of cancer-associated deaths worldwide according to the global cancer statistics [1,2]. Despite great advancements in the diagnostic methods and treatment of gastric cancer, the 5-year overall survival rate of gastric cancer patients is still low, less than 25%, due to the high tendency of lymphatic metastasis [3]. In consequence, illuminating the molecular mechanism underlying gastric cancer invasion and metastasis is essential for accelerating progress in the development of effective treatments and targeted drugs for GC patients. In recent years, different non-coding RNAs (ncRNAs), which are largely classified as microRNA (miRNA), long non-coding RNA (lncRNA), and PIWI-Interacting RNA (piRNA), have been found to play a vital role in critical signaling pathways of gastric cancer carcinogenesis [4–7]. Besides, a particular kind of ncRNAs, called circular RNA (circRNA), has attracted increasing attention in a variety of tumors research and several pieces of evidence point to that circRNA played a role in the development of gastric cancer [8,9]. Hence, it is necessary to have a more extensive and in-depth exploration of this exciting field of circRNA in gastric carcinoma.

CircRNAs were first discovered under an electron microscope by Hsu and Coca Prados in 1976 [10]. CircRNAs are formed by back-splicing of the exons or introns – generated transcript of the parent gene. Compared with linear non-coding RNA, the circRNA molecule has a continuous closed circular structure, and its covalent closed-loop structure lacks a 5ʹ to 3ʹ polarity and a polyadenylated tail and is resistant to RNA exonucleases or RNase R digestion, so it has a high degree of stability in different species [11]. With the development of reliable bioinformatics and high-throughput sequencing technology, vast circRNAs have been identified to be abundant, diverse, and conserved molecules in mammalian cells and tissues [12]. Mounting evidence has uncovered that circRNAs are involved in transcriptional and post-transcriptional gene expression, and abnormal expression of circRNAs is associated with different types of tumorigenesis and progression by regulating a variety of biological processes, including proliferation, invasion, and metastasis [13].

The function of some circRNAs in gastric cancer has also been studied to some extent. For example, circCOL6A3_030 (circBase ID: hsa_circ_0006401; circRNADb ID: hsa_circ_28198; circBank ID: hsa_circCOL6A3_030) is derived from the reverse splicing of COL6A3 mRNA (from 3 exons (exon 4–6)) on chromosome 2q37.3, with a total length of 761 nucleotides (circRNADb website). The literature showed that in vitro experiments, the over-expressed circCOL6A3_030 promoted the proliferation, migration, and apoptosis of gastric cancer cells by relieving the inhibition of mir-3064-5p on COL6A3 [14].

The purpose of this study was to investigate the expression, function and molecular mechanism of circCOL6A3_030 in gastric cancer, and to discover its poly-peptide encoding function. Specifically, we found that circCOL6A3_030 was highly expressed in GC tissues with lymph node metastasis. In vitro, silencing circCOL6A3_030 inhibited GC cell migration, but had no significant effect on proliferation. In vivo, silenced-expression of circCOL6A3_030 could reduce liver metastasis of tumors. Mechanistically, we demonstrated that circCOL6A3_030 may promote metastasis by encoding proteins. It is hoped that our study can provide a deeper understanding of gastric cancer and find a new effective therapeutic target for gastric cancer.

## Materials and methods

### Patients and clinical tissue samples

This work was conducted under the approval of the Research Ethics Committee of Zhejiang Provincial People’s Hospital, Hangzhou Medical College (code: 2020QT084). All human researches were based on the Helsinki Declaration. Each participant had provided written informed consent before enrollment.

Forty-one tissue specimens and paired adjacent non-cancerous tissue samples, which obtained from GC patients who underwent radical resection of the primary lesions at Zhejiang Provincial People’s Hospital, Hangzhou Medical College, were collected from May 2017 to July 2018 (Table 1). All Patients without any preoperative radiotherapy, chemotherapy, or any other medical interventions were diagnosed as primary GC by postoperative pathological examination. Non-cancerous tissue was obtained 5 cm from the tumor site. Immediately after removal, all samples were frozen in sterile liquid nitrogen test tubes and stored at −80°C for further analysis. The tumor stage was accurately defined according to the eighth edition of the tumor–node–metastasis (TNM) classification system of the International Union Against Cancer (UICC, 2009). Two experienced pathologists assessed the histological grade. Detailed clinical and pathological data, including age, sex, diameter, differentiation, lymphatic metastasis, and TNM stage, were available for all patients (Table 1).

**Table 1.** Clinical characteristics of the patients

Characteristics	Validation
Total number	41
Gender (male*/*female)	31/10
Age (years, mean)	65.1 ± 11.2
Primary GC (yes*/*no)	41/0
Adenocarcinoma (yes*/*no)	
– Tumor size T ≤ 2 cm	41/0
– T > 2 cm	0/41
Number of lymph nodes	
−0	21
−1-2	6
−3-6	6
-≥7	8
TNM stage	
– I	17
– II	14
– III	9
– IV	1
Histologic differentiation	
Well differentiated	6
Moderately differentiated	17
Poorly differentiated	18
Vascular invasion (yes/no)	20/21

*GC: Gastric cancer; TNM: Tumor, node (regional lymph node), metastasis*

### Microarray data collection

The microarray data used in this study were obtained from the public GEO (Gene Expression Omnibus) database (https://www.ncbi.nlm.nih.gov/geo/). The circRNA expression profiles of GC were acquired from GSE100170. The raw microarray data were then background corrected, normalized, and log2-transformed. The Bio-conductor Limma package was used to screen for differential expression of circRNAs (DEcircRNAs) between the gastric cancer lymph node metastasis group and the non-lymph node metastasis group in the environment of R-3.6.1, and the cutoff value was set at the adjusted P-value of < 0.05 and |log2 fold change (FC)| >2.0 [15]. The circBase (http://www.circbase.org/) and the circBank (http://www.circbank.cn/index.html) were used to find the original gene associated with the circRNA. The RNA-sequencing (RNA-seq) data were downloaded from the public TCGA (The Cancer Genome Atlas) data portal (https://tcga-data.nci.nih.gov/tcga/).

### Cell line, cell culture, and transfection

Normal human gastric epithelium cell (GES-1), highly differentiated cell MGC-803, medium differentiated cell SGC-7901, poorly differentiated cell BGC-823, AGS cell, and HGC-27 cell lines, stored in our laboratory, were purchased from the Cell Bank of the Shanghai Institute of Biochemistry and Cell Biology, where they were tested and authenticated according to American Type Culture Collection standards. These cell lines were cultured in RPMI-1640 or DMEM media (Gibco) supplemented with 10% fetal bovine serum (FBS, Gibco) and 1% penicillin/streptomycin mixture (Selleck, China) and maintained at 37°C in a humidified atmosphere of 5% CO_2_ and 95% air. Exponentially growing cells were harvested from the culture flasks using 0.25% Trypsin/EDTA (Beyotime, China), and centrifuged at 1000 rpm for 5 min, re-suspended, and counted for use in subsequent experiments.

Lipofectamine® 2000 reagent (Invitrogen, #11,668-027) was used to transfect cells with constructed plasmids (GenePharma, Shanghai) and small interfering RNAs (siRNAs) (RiboBio, Guangzhou), as following the instructions provided by the manufacturer. Human GC cells in logarithmic growth period cultured on the 6-well plate (2.0 × 10^5^cells/well) were respectively transfected with 2 µg circCOL6A3_030 + GFP plasmid, circCOL6A3_030 -ORFmut + GFP plasmid, empty vector (EV), 50 nM negative control (si-NC), and siRNAs of circCOL6A3_030 1#, 2#, and 3 #. circCOL6A3_030+ GFP plasmid and circCOL6A3_030-ORFmut+GFP plasmid were synthesized by GenePharma.

The siRNA sequences were as follows:

si-circCOL6A3_030 1#:5ʹ-ACCTGTAATAACCTTCTGCAAUU-3ʹ;

si-circCOL6A3_030 2#:5ʹ-AGACCTTCTGTATCTGACCAAUU-3ʹ;

si-circCOL6A3_030 3#:5ʹ-TAACAATCCTCCTGTACCTAAUU-3ʹ.

Transfection efficiency was verified by real-time quantitative polymerase chain reaction (qRT-PCR) 48 h later.

### RNA extraction, purification, and qRT-PCR

Total RNA was extracted from cancerous/noncancerous specimens (stored at −80°C) and cells using Trizol (Invitrogen, USA) as per manufacturer’s protocol. The concentration, purity and integrity of RNA were determined by NanoDrop spectrophotometer OD260/280 and 1% formaldehyde-denatured gel electrophoresis. Then, RNA was purified and reverse transcribed into cDNA using the SuperScript™ IVFirst-Strand Synthesis System (Invitrogen, USA). SYBR^TM^ Green PCR Master Mix was used to detect the expression levels of circRNAs and the mRNAs by qRT-PCR in ABI 7500 real-time PCR system. The reaction conditions were according to the manufacturer’s protocol (Thermo Fisher Scientific, USA). We used the human GAPDH reference gene as internal controls. Relative transcription expression value was estimated using a 2− comparative Ct (2− ΔΔCt) method and normalized against the threshold cycle (Ct) of GAPDH. All reactions were conducted in triplicate independently to ensure the reproducibility of all the data. The primer sequence information (RiboBio, China) is presented as follows:

circCOL6A3_030 (Forward, 5ʹ-TGGCTCTCACTGAAACAGAAATG-3ʹ;

Reverse, 5ʹ-GTCGTCAC TGGGTTGGATGTAG-3ʹ),

LineCOL6A3_030 (Forward, 5ʹ-ATGAGGAAACATCGGCACTTG-3ʹ;

Reverse,5ʹ-GGGCATGAGTTGTAGGAAAGC-3ʹ),

GAPDH (Forward, 5ʹ- AGTCAGCATT TCACAAGACCTC-3ʹ;

Reverse, 5ʹ- CAGGCGAAGATGTTCTGGC-3ʹ)

### circRNA confirmatory assay

The divergent primers were synthesized by RiboBio (Guangzhou, China) to verify the back-splicing junction of circCOL6A3_030. Sanger sequencing (GENESEED, Guangzhou, China) of the cDNA PCR product bands (Invitrogen, USA) was performed to further determine the full-length and ensure the accuracy of the circRNA detection.

### Fluorescence in situ hybridization (FISH)

RNA fluorescence in situ hybridization (RNA-FISH) was performed with FISH Kits according to the manufacturer’s instructions (RiboBio, China) in gastric cells. RNA FISH probe sequence targeting the back-splicing site of circCOL6A3_030 was designed and synthesized by RiboBio (Guangzhou, China). First, cells were seeded on coverslips, fixed with 4% paraformaldehyde for 10 min, and then permeabilized with 0.5% Triton X-100 PBS for 5 min. Next, hybridization was carried out with a Cy3-labeled FISH probe in a dark moist chamber at 37°C for 16 h. Afterward, slides were washed in hybridization rinse solution and were stained with 4,6-diamidino-2-phenylindole to label cell nuclei (DAPI; Life Technologies, USA) for 10 min. All images were obtained using a confocal microscopy (Leica, Germany).

### Cell counting assay

SGC-7901 and BGC-823 cells were transfected with 50 nM si-circCOL6A3_030 1#, 2#, 3 #, or si-NC in a 6-well plate. Cells were collected 24 h after transfection and placed in a 6-well plate in the form of 2 × 10^5^ cells/wells, with three duplicate wells of each group. The number of cells was counted after 2 days.

### Transwell assay

SGC-7901 and BGC-823 cells were transfected with 2 µg circCOL6A3_030 + GFP plasmid, circCOL6A3_030 -ORFmut +GFP plasmid, empty vector (EV), 50 nM negative control (si-NC), and siRNAs of circCOL6A3_030 1#, 2#, and 3 # in a 24-well plate. After transfection for 24 h, transfected cells were harvested, and then carried out migration experiments. For migration assays, 1 × 10^5^ transfected cells were placed in a Transwell (24-well, Millipore, USA) upper chamber with 200 μl of serum-free RPMI 1640, while 600 μl RPMI 1640 containing 10% FBS was added to the bottom chamber as a chemical attractant. At 24 h of post-incubation, the chamber was fixed with polyformaldehyde for 30 mins and stained with a 0.1% crystal violet solution for 15 mins, and then the cells on the inner surface of the upper chamber were removed by scrubbing with a sterile cotton swab dipped in PBS. After that, five fields were randomly selected under an inverted microscope (Olympus, Tokyo, Japan) to count the average cell number in the lower chamber (200 x). The group under each condition had its duplicate wells.

### Scratch wound assays

SGC-7901 and BGC-823 cells were transfected with 2 µg circCOL6A3_030 + GFP plasmid, circCOL6A3_030 -ORFmut +GFP plasmid, empty vector (EV), 50 nM negative control (si-NC), and siRNAs of circCOL6A3_030 1#, 2#, and 3 # in a 6-well plate. After 24 h infection, transfected cells were collected, and then subjected to a wound-healing assay. For wound-healing assays, the transfected cells were plated in 6-well plates (4 × 10^5^ cells /well). At 24 hours after inoculation, wounds were created by using a 1 ml pipette tip in confluent cells with reaching approximately 80%. The free-floating cells and debris were then washed out with PBS. The cells were incubated at 37°C with RPMI 1640 medium containing 2% FBS. Wound healing was observed at different time points and scrape lines were photographed at the same time. The group under each condition had its duplicate wells, and each experiment was repeated three times.

### Prediction of circCOL6A3_030 encodes polypeptide

The circRNADb was used to gather evidence of the circRNA ability to encode proteins (http://reprod.njmu.edu.cn/cgi-bin/circrnadb/circRNADb.php). There were circCOL6A3_030 IRES and ORFs information. Bioinformatic metrics, such as the IRES score and ORF score are used to assess the coding potential of detected IRESs and ORFs (Table 2).

**Table 2.** Bioinformatic metrics of protein coding potential

Item	Parameter Index		
IRES Elements	Position (start–end)	R Score	With Pseudoknot (Y/N)
	513–649	1.686954	Y
	233–380	1.588748	Y
Open Reading Frame (ORF)	Start Position	End Position	Protein Length
	212	1 r + 47	198 aa
	MATVRPPVAV KPATAAKPVA AKPAAVRPPA AAAAKPVATK PEVPRPQAAK PAATKPATTK PMVKMSREVQ VFEITENS**AK LHWERAEPPG PY**FYDLTVTS AHDQSLVLKQ NLTVTDRVIG GLLAGQTYHV AVVCYLRSQV RATYHG**SFST KKSQPPPPQP** ARSASSSTIN LMVSTEPLAL TETEMFRITL LQVLHPTQ*		
	Note: (1). nr represents n rounds(n < 3); (2). * represents a stop codon.		

The data comes from the website of circRNADb. (http://reprod.njmu.edu.cn/cgi-bin/circrnadb/circRNADb.php)

### Western blotting

Total protein was extracted from cells using RIPA (Radio-Immunoprecipitation Assay) buffer (Sigma) with a 1% protease inhibitor and 1% PMSF (Beyotime Biotechnology, China). Protein concentrations were measured using the BCA protein assay kit (Beyotime Biotechnology, China). Proteins were denatured with sodium dodecyl sulfate (SDS) buffer at100°C for 10 min, and each sample protein of 30 μg then was segregated by SDS-polyacrylamide gel electrophoresis (SDS-PAGE). The separated protein bands were transferred onto polyvinylidene fluoride (PVDF) membrane (Millipore, USA). Next, the PVDF membrane was blocked with 5% nonfat milk in TBST buffer for 1 h at room temperature and then were incubated with specific primary antibodies at 4°C overnight including circCOL6A3_03 0 peptide (1:50; HuaAn Biotechnology Co., Ltd, China) and the internal reference protein antibody GAPDH (1:5000; Cell Signaling Technology, USA). Afterward, the PVDF membrane was washed three times for ten  mins in TBST and incubated with Horseradish peroxidase (HRP)-conjugated goat anti-rabbit IgG secondary antibodies (1:1500; Cell Signaling Technology, USA) for 2 h at room temperature. After washing in TBST for 3 times, immunoblot bands were visualized by ECL chemiluminescent reagent (Millipore, USA) under a Bio-Rad XRS chemiluminescence detection system (Bio-Rad, USA) and the optical density was analyzed by ImageJ2X software. These experiments were repeated three times.

The peptide antibody was designed and synthesized by HuaAn Biotechnology Co., Ltd (Hangzhou, China). The peptide sequence of circCOL6A3_030 was as follows: 147–161aa CSFSTKKSQPPPPQP; 79–92aa AKLHWERAEPPGPYC.

### Xenograft model

The procedures for care and use of animals were approved by the Principles of Laboratory Animal Care of Zhejiang Chinese Medical University and Zhejiang Provincial People’s Hospital. All animal studies were performed following the Guidelines for the Care and Use of Laboratory Animals of the Council of Science and Technology of China.

**Figure 1.**
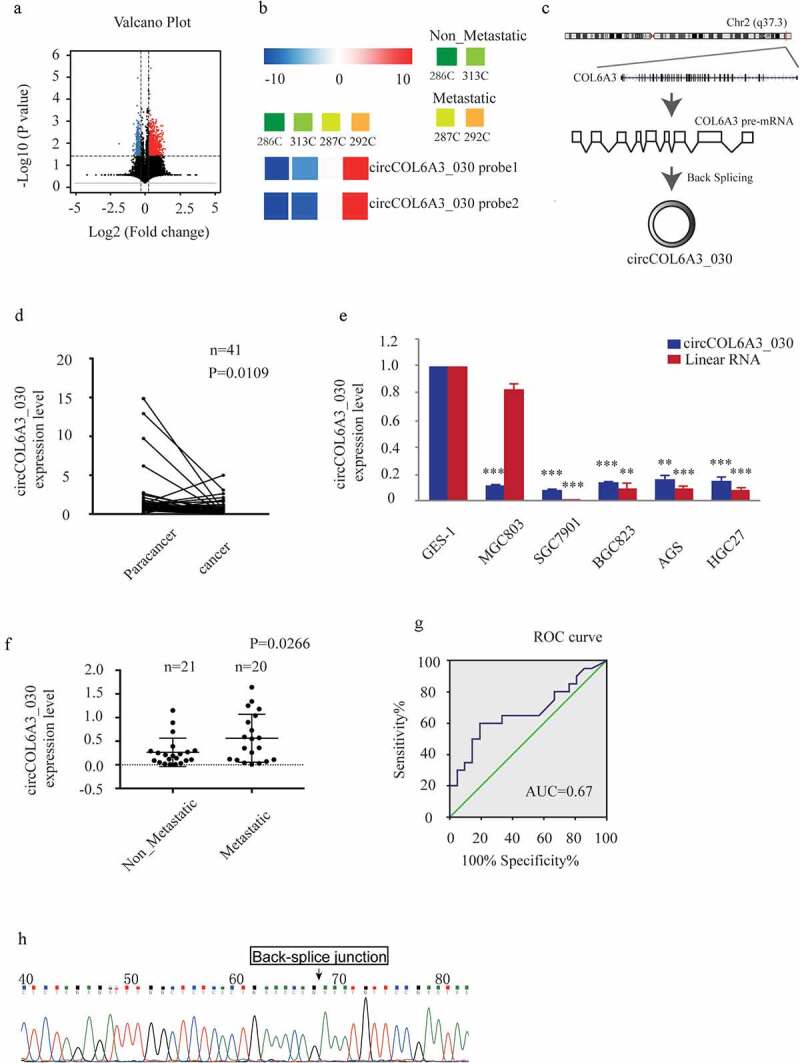
circCOL6A3_030 was up-regulated in GC tissues with lymph node metastasis. A. In the GSE100170 database, volcano plot shows the distribution of DECs (differentiated expressed circRNAs) between the two groups (GC tissues without lymph node metastasis group and GC tissues with lymph node metastasis group). B. In the GSE100170 database, the heat map showed the expression of circCOL6A3_030 in different tissues (286 C and 313 C are GC tissues without lymph node metastasis group; 287 C and 292 C are GC tissues with lymph node metastasis group). C. circCOL6A3_030 (hsa_circ_0006401, 761bp) genome location and formation diagram. D-F. The expression levels of circCOL6A3_030 in gastric cancer tissues and cell lines were detected by qRT-PCR. G. ROC curve was used to evaluate the diagnostic value of circCOL6A3_030 for gastric cancer metastasis (AUC = 0.675; sensitivity, 92.5%; and specificity, 77.5%). H. Sanger sequencing was performed to indicate circCOL6A3_030 with a back spliced junction. (*p < 0.05; **p < 0.01; ***p < 0.001; GC: Gastric cancer)

For the in vivo assay of a gastric cancer mouse model with spleen-to-liver metastasis, thirty-six female (4-week-old) BALB/c nude mice (20 ± 2 g) were purchased from the Shanghai SLAC Laboratory Animal Co.Ltd (Shanghai, China) and maintained in SPF condition with a standard 12-h light-dark cycle at the Experimental Animal Center of the Zhejiang Chinese Medical University. On the day of the intrasplenic injection of liver metastases, SGC-7901 cells transfected with si-circCOL6A3_030 3# and si-NC (Ctrl) were harvested by trypsinization and washed twice with serum-free medium and then suspended in PBS (1 × 10^6^/100 μl in serum-free medium with 50% Matrigel). Cells were injected into the superior pole of the mouse spleen (Figure 1a). The mice were anesthetized with 4% chloral hydrate (10 ml/kg) by peritoneal injection on day 21 after intrasplenic injection with the tumor cell lines. The livers were removed and the hepatic metastatic nodules were observed. Liver tissues were fixed in 10% buffered formaldehyde solution for hematoxylin and eosin (H&E) staining and immunohistochemical Staining.

### Hematoxylin and eosin staining and immunohistochemical staining

#### Hematoxylin and Eosin Staining

At room temperature, the specimens were fixed in 4% paraformaldehyde for 24 hours, followed by dehydration and embedding in paraffifin. After dewaxed and rehydrated, the 5-μm-thick tissues were stained with hematoxylin and eosin (H&E).

#### Immunohistochemical Staining

The specimens were blocked with 5% normal goat serum for 30 minutes and then incubated with primary antibody against circCOL6A3_030 (1:50; HuaAn Biotechnology Co., Ltd, China) at 4°C overnight. Then, sections were processed using the ABC detection kit (Vector Laboratories, Burlingame, CA). All the staining images were obtained using the Olympus microscope (Olympus Co., Tokyo, Japan)

## Statistical analysis

Each experiment was repeated in triplicate. Statistical analyses were carried out by SPSS 21.0 software (SPSS, USA). The circRNA expression level was presented as an FC utilizing the 2-ΔΔCT method on qRT-PCR analysis. Differences between individual groups were analyzed using Student’s t-test. All data were expressed as the mean ± standard deviation (SD). A p-value of < 0.05 (*), < 0.01 (**) or < 0.001 (***) was considered significant.

## Results

This study attempted to investigate the mechanism of circCOL6A3_030 in the development of gastric cancer. High levels of circCOL6A3_030 were observed in gastric cancer with lymph node metastasis. In vitro scratch and Transwell experiments showed that circCOL6A3_030 knockout inhibited cell migration. In vivo studies showed that circCOL6A3_030 knockdown reduced liver metastasis of tumors. CircCOL6A3_030 could encode a small peptide, the presence of which was confirmed by immunohistochemistry, and the small peptide promoted cell migration by in vitro scratch assay, suggesting that circCOL6A3_030 may promote the migration of gastric cancer cells by encoding a small peptide.

### circCOL6A3_030 was up-regulated in GC tissues with lymph node metastasis

circCOL6A3_030 is formed by the circulation of the exons (4–6) of COL6A3 gene located on chromosome 2 (q37.3) (Figure 1c). GC-related microarray data were obtained from the NCBI Gene Expression Omnibus Database (GEO), and differential circRNAs between the gastric cancer lymph node metastasis group and the non-lymph node metastasis group were calculated (Figure 1a). We found that the expression of circCOL6A3_030 was up-regulated in gastric cancer tissues with lymph node metastasis compared with those without lymph node metastasis (Figure 1b). To further confirm the expression of circCOL6A3_030 in GC, we performed qRT-PCR between 41 pairs of GC tissues and their paired normal tissues. The results showed that circCOL6A3_030 was down-regulated in gastric cancer tissues and cell lines compared with the paired normal gastric tissue (Figures 1D and 1E). More interestingly, circCOL6A3_030 was up-regulated in GC with lymph node metastasis (Figure 1f). Moreover, the diagnostic potential of circCOL6A3_030 was also determined by receiver operating characteristics (ROC) curve analysis. We found that circCOL6A3_030 provided high diagnostic power for detection of lymph node metastasis of gastric cancer (AUC = 0.675; sensitivity, 92.5%; and specificity, 77.5%) (Figure 1g). In addition, in order to further verify our results, PCR products were sequenced and identified as circRNAs with a back spliced junction (Figure 1h).

### circCOL6A3_030 increased migration of GC in vitro

**Figure 2.**
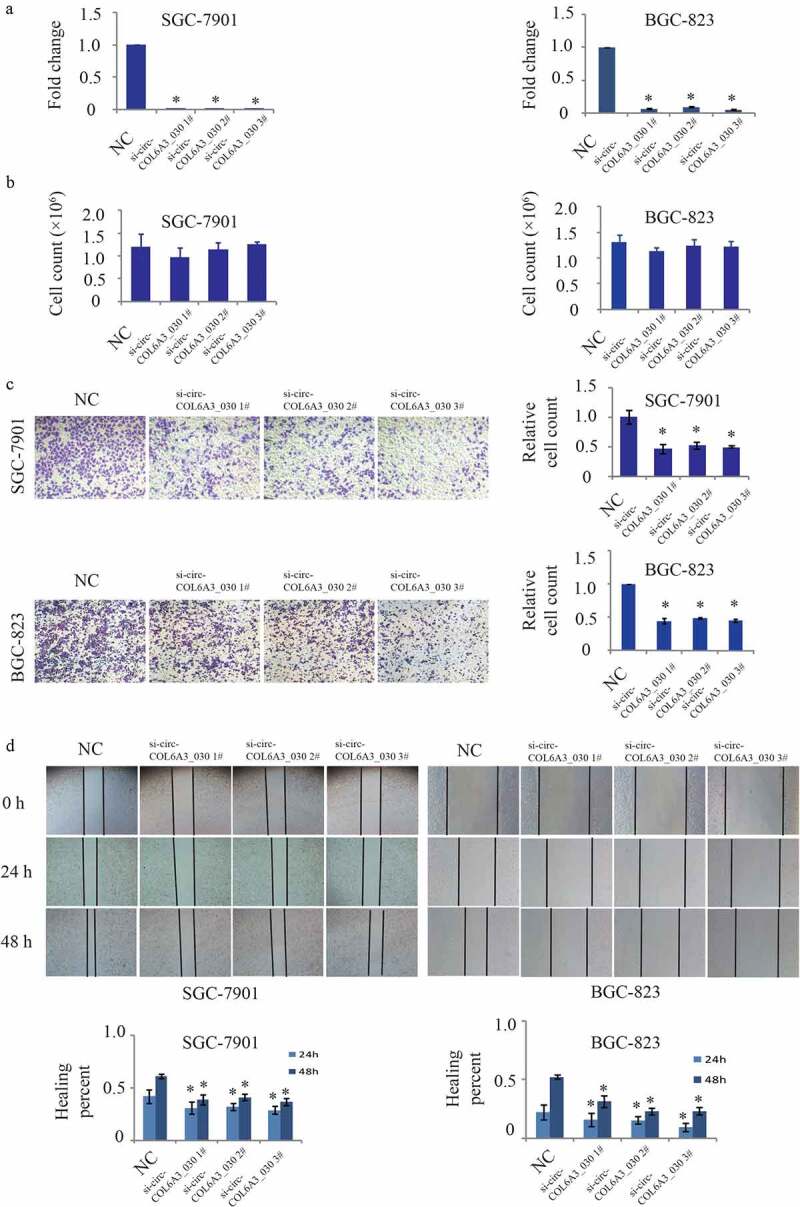
circCOL6A3_030 increased the migration of GC in vitro. A. The interference efficiency of three small interfering RNAs targeting circCOL6A3_030 in gastric cancer cells. B. There was no significant difference between the proliferation rate of different groups in SGC-7901 and BGC-823 cell lines. C-D. Compared to the negative control, migration of circCOL6A3_030 silenced GC cells were significantly inhibited in both SGC-7901 and BGC-823 cell lines transwell assay (2 C) and wound-healing assay (2D). (*p < 0.05; **p < 0.01; ***p < 0.001)

To study the biological function of circCOL6A3_030, three siRNAs were designed according to the splice junction of circCOL6A3_030. As shown in Figure 2a, circCOL6A3_030 was perfectly down-regulated by si-circCOL6A3_030 1#, si-circCOL6A3_030 2# and si-circCOL6A3_030 3# in SGC-7901 and BGC-823 cell lines. To investigate the role of circCOL6A3_030 on the proliferation of GC, 2 × 10^5^ cancer cells transfected or not transfected with circCOL6A3_030 siRNA were cultured for 2 days, and the number of cells was then counted. There was no statistically significant difference in proliferation rate between the groups (Figure 2b). Moreover, the effect of circCOL6A3_030 on GC cells migration was investigated by transwell assay (Figure 2c) and wound-healing assay (Figure 2d). The results showed that compared to the negative control, migration of circCOL6A3_030 silenced GC cells were significantly inhibited in both SGC-7901 and BGC-823 cell lines (Figure 2c-d).

### circCOL6A3_030 increased GC metastasis in vivo

**Figure 3.**
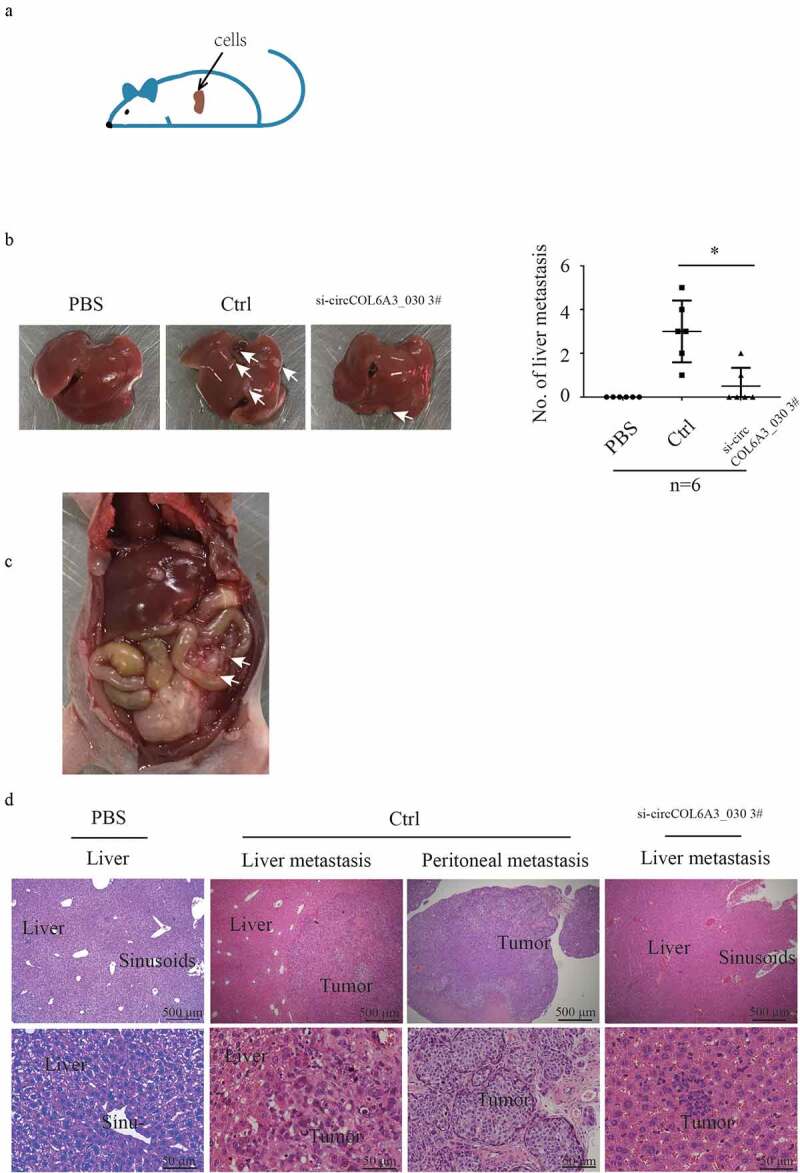
circCOL6A3_030 increased GC metastasis in vivo. A. Schematic diagram of in vivo experimental model:cells were injected into the superior pole of the mouse spleen into nude mice. **B**. After 3 weeks of injection, the mice were dissected to find gastric cancer metastases in the liver. **C**. Representative peritoneal dissemination of SGC7901 cells in negative control transfected nude mice group. **D**. H&E stain of the primary tumor and metastatic lesions. (*p < 0.05; **p < 0.01; ***p < 0.001)

To further evaluate the function of circCOL6A3_030 in vivo, negative control and circCOL6A3_030 silenced SGC-7901 cells were injected into the superior pole of the spleen of nude mice (Figure 3a). Mice were sacrificed 3 weeks after cell injection, peritoneum was observed, and whole liver tissues and tumor tissues of mice were collected for fixation. H&E staining was used to assess the number of liver metastases in gastric cancer in mice. As shown in Figure 3b, the number of mice with liver metastasis in the circCOL6A3_030 silenced group was decreased by 50% compared with the control group. Moreover, peritoneal metastasis nodes were observed in two of six negative control transfected nude mice (Figure 3c). However, there were no peritoneal metastasis nodes in circCOL6A3_030 silenced group and PBS injecting nude mice group. The H&E staining of the primary tumor and metastatic lesions were exhibited in Figure 3d.

### circCOL6A3_030 encoded a 198aa peptide

**Figure 4.**
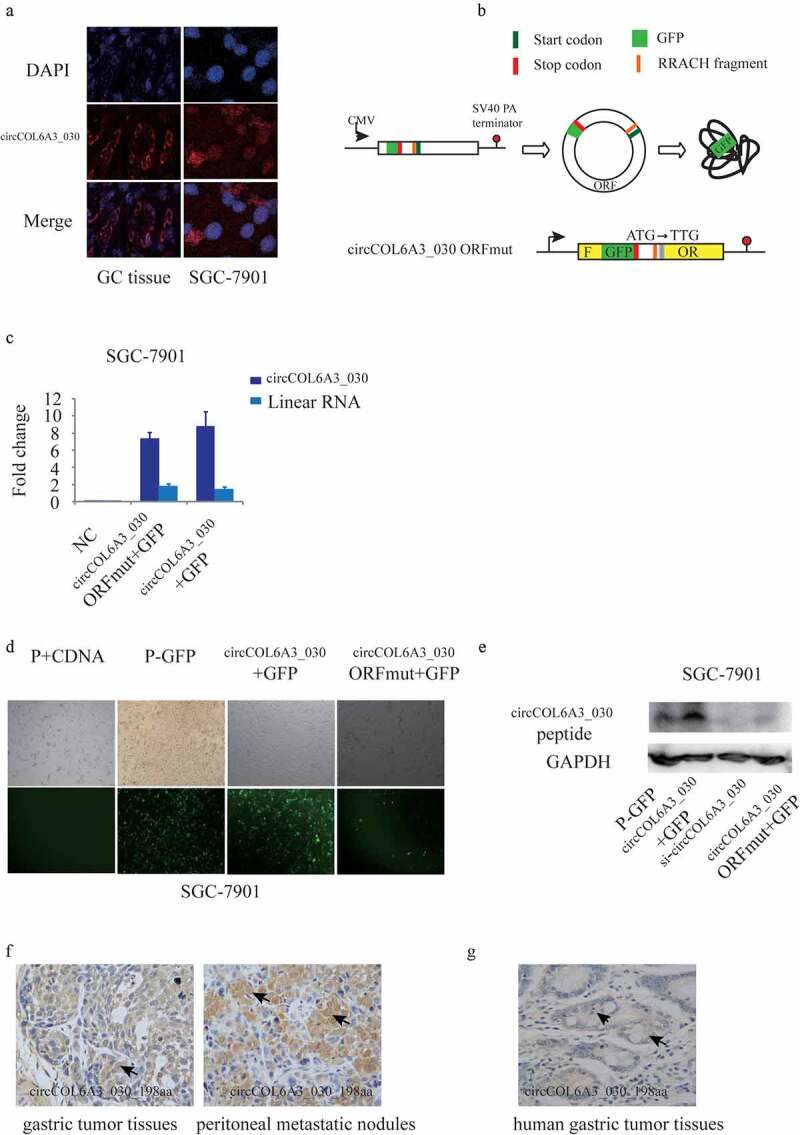
circCOL6A3_030 encoded a 198aa peptide. A. RNA-FISH staining: circCOL6A3_030 was distributed in both nucleus and cytoplasm. B. Module diagram of the construction of the vector of circCOL6A3_030 + GFP and circCOL6A3_030-ORFmut + GFP. C. qRT-PCR results showed that ORF mutation didn’t affected circCOL6A3_030 expression. D. The image under a fluorescence microscope: fluorescence could be observed in the circCOL6A3_030 + GFP group, but hardly in the circCOL6A3_030-ORFmut + GFP group. E. Western blot analysis was used to detect the expression of circCOL6A3_030 peptide. F. Immunohistochemistry was used to detect the expression of 198aa polypeptide in gastric tumor tissues of mice, peritoneal metastatic nodules of mice and human gastric cancer tissues

To further investigate the mechanism of circCOL6A3_030 to regulate GC metastasis, RNA-FISH staining of GC tissues and SGC-7901 cell line was carried out. We found that circCOL6A3_030 was distributed in both nucleus and cytoplasm (Figure 4a). Recent studies showed that in some conditions, circRNAs can be translated into protein or peptide in a splicing-dependent and cap-independent manner. Moreover, circRNA-derived protein played important biological functions in varied cell processes. circCOL6A3_030 originates from the circularization of the fourth to the sixth exon of its host gene. A 597-nt open reading frame (ORF) was present in circCOL6A3_030, spanning from the putative AUG (Start Position: 212) of the host gene to a STOP codon (End Position: 1 r + 47). To determine whether this ORF (circCOL6A3_030-ORF) was functional, We used an expression vector capable of producing circular transcription. The vector contained the fourth to the sixth exon of the COL6A3 gene and was able to express circCOL6A3_030 at high levels (Figure 4b). Construct a p-circ vector containing a GFP labeled sequence directly upstream of the circRNA sequence termination codon, so that GFP labeled proteins can only be produced after the formation of a circular template (P-GFP). qRT-PCR results showed that ORF mutation didn’t affected circCOL6A3_030 expression (Figure 4c). The Figure 4d showed the results that the circCOL6A3_030 + GFP plasmid-expression group showed more fluorescence than p-GFP group and the circCOL6A3_030 -ORFmut +GFP group not (Figure 4d). To detect the peptide encoded by circCOL6A3_030 in cells, we produced antibodies against the circCOL6A3_030 peptide. The circCOL6A3_030 peptide was detected using an anti-circCOL6A3_030 antibody in circCOL6A3_030 + GFP plasmid-transfected, but mutation of the circCOL6A3_030 ORF start codon abolished the detection of the circCOL6A3_030 peptide (Figure 4e). Immunohistochemistry suggested that 198aa polypeptide was expressed in mice gastric tumor tissues and peritoneal metastatic nodules (Figure 4f). Immunohistochemistry also indicated the presence of circCOL6A3_030_198aa in human gastric cancer tissues (Figure 4g). Taken together, these data indicated that circCOL6A3_030 may regulate the metastasis of GC by encoding a 198aa peptides.

### circCOL6A3_030 peptide, Not circCOL6A3_030, promoted GC metastasis

**Figure 5.**
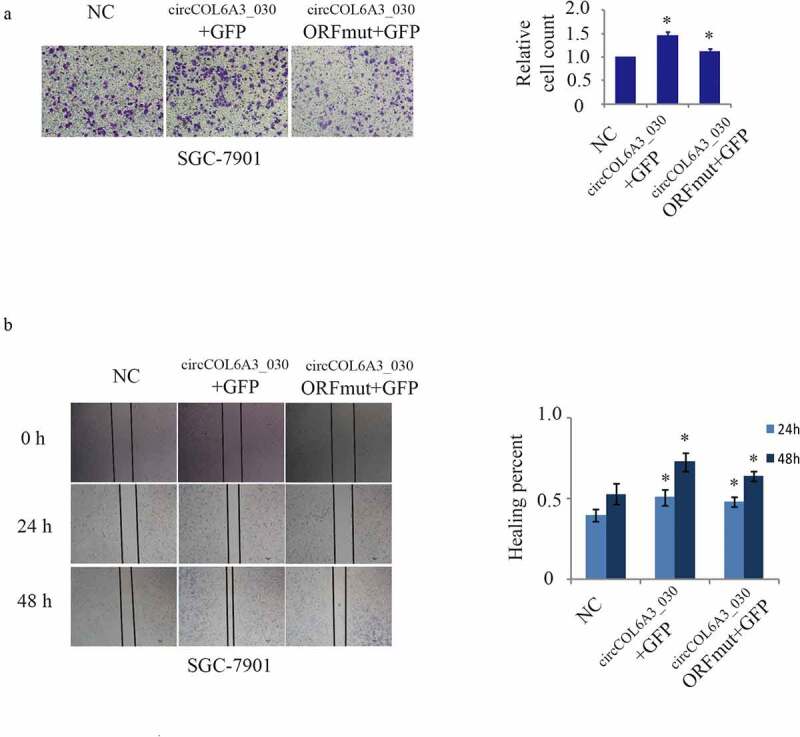
circCOL6A3_030 peptide, Not circCOL6A3_030, prompted GC metastasis. A. Compared to negative control, SGC-7901 cell lines migration of circCOL6A3_030 + GFP group were significantly enhanced in transwell assay. B.Compared to negative control, SGC-7901 cell lines migration of circCOL6A3_030 + GFP group were significantly enhanced in wound-healing assay. (*p < 0.05; **p < 0.01; ***p < 0.001; GC: Gastric cancer)

To investigate the influences of the circCOL6A3_030 peptide and circCOL6A3_030 on GC metastasis, the circCOL6A3_030 + GFP plasmid-expression and circCOL6A3_030 -ORFmut +GFP plasmid constructs were transfected into GC cells. The circCOL6A3_030 + GFP plasmid-expression, which expresses the circCOL6A3_030 peptide, promoted cancer cell migration (Figure 5a-b). In contrast, the circCOL6A3_030 -ORFmut +GFP plasmid, which contained a mutated circCOL6A3_030 start codon and therefore didn’t encode the circCOL6A3_030 peptide, so cell migration of the circCOL6A3_030-ORfmut +GFP group was inhibited compared with the circCOL6A3_030 + GFP group (Figure 5a–B). Moreover, as shown in Figure 4c, qRT-PCR results showed that ORF mutation didn’t affect circCOL6A3_030 expression. Collectively, these data showed that circCOL6A3_030 encoded a small peptide that had a founction as a tumor-promoting factor.

## Discussion

The role of circRNAs in gastric cancer has been attracting more and more attention. Meanwhile, the gradually accumulated microarray data related to circRNAs of gastric tumor also provide a lot of information for our research. Microarray data were screened out candidate circRNAs by recognizing significantly different expressions [16]. In the present study, among the significantly differentially expressed circRNAs of the tissues of gastric cancer with and without lymph node metastasis, the up-regulation of circCOL6A3_030 caught our attention. The host gene of circCOL6A3_030 was COL6A3 (collagen VI alpha 3), encoding the α-3 chain of type VI collagen, which has been found to participate in the tumor malignant processes in gastric, pancreatic, and ovarian cancer [17]. So we hypothesized that circCOL6A3_030 also played an important role in the malignant progression of gastric cancer.

Up-regulation of circCOL6A3_030 in gastric cancer with lymph node metastasis were then further validated by detecting forty-one of GC tissues. At the same time that we were doing this, Xiaoli Sun team study showed that over-expressed circCOL6A3 promoted cell proliferation, migration, and apoptosis of gastric cancer through the rescission of miR-3064-5p-induced inhibitory effect on COL6A3 [14]. Consistent with their results, depleted circCOL6A3 restricted migration of GC cells in vitro. Also, we further proved that silencing circCOL6A3_030 could inhibit liver metastasis of gastric cancer cells in vivo experiments.

In terms of function, numerous studies have revealed that circRNAs had abilities of microRNA sponges, circRNA-protein interaction, transcription and splicing regulation, translation, and other [18]. Sponging miRNAs may be the most common mechanism of action for some circRNAs which derived from exon (ecircRNA). For example, Zai Luo et al. found that circCCDC9 served as an endogenous competing RNA and was bonded to miR-6792-3p to affect CAV1 expression to suppress the progression of gastric cancer [19]. CircRNAs and RNA-binding proteins (RBPs) are also considered as basic elements of circRNAs function. For instance, Yan-Jing Zhu et al. reported that circZKSCAN1 inhibits the binding of FMRP to the β-catenin-binding protein-cell cycle and apoptosis regulator 1 (CCAR1) mRNA by competitive binding of FMRP, thereby inhibiting the transcriptional activity of Wnt signal [20]. Introncontaining circRNAs (ciRNAs or ElciRNAs), predominantly localized in the nucleus, regulates parental gene transcription through cis-regulatory mechanisms. Zhaoyong Li hinted that EIciRNAs could interact with U1 snRNP to promote the transcription of their parent genes by binding RNA Pol II at the promoter region of the parental gene [21]. Contrary to previous studies, recent studies have shown the potential of circRNAs to participate in translation. The mechanisms of circRNAs translation that have been discovered so far are as follows: internal ribosome entry site (IRES) and open reading frame (ORF) mediated translation, rolling circle amplification (RCA), and m6A modified-driven translation [22]. There is also evidences that circRNAs can successfully translate GFP in E.coli by inserting GFP (green fluorescent protein) into circRNA with an open reading frame [23,24]. Furthermore, it has been studied that some circRNAs translations are involved in the regulation of their host gene function. Wei-Cheng Liang found that β-catenin-370aa protects β-catenin from the degradation of the ubiquitin-proteasome by competitively binding to GSK3β [25].

As for our study, we preliminarily investigated the translation function of circCOL6A3_030, and the results showed that circCOL6A3_030 had the function of encoding short peptide, Meanwhile, in vitro experiments proved that the reduction of small peptide synthesis can inhibit migration, which will provide important clues for exploring the mechanism of promoting gastric cancer metastasis.

## Conclusions

The above studies proved that circCOL6A3_030 expression was up-regulated in tumor tissue samples with lymph node metastasis, and circCOL6A3_030 coding peptide was confirmed to regulate gastric cancer invasion in vitro and in vivo, which provided a new direction for us to study the role of circCOL6A3_030 in the process of gastric cancer metastasis.

## Limitation

In future studies, we need more specimens to determine the expression of circCOL6A3_030 encoded small peptides in tissues of gastric cancer patients.

## Highlights

1. The up-regulation of circCOL6A3_030 is associated with lymph node metastasis in gastric cancer.
2. circCOL6A3_030 can be used as a good marker for lymph node metastasis in gastric cancer.
3. The down-regulation of circCOL6A3_030 inhibits the migration of gastric cancer cells and reduces the liver metastasis of gastric cancer cells.
4. circCOL6A3_030 promotes GC cell migration by encoding a small peptide, circCOL6A3_030_198aa.
